# Questionable lonsdaleite identification in ureilite meteorites

**DOI:** 10.1073/pnas.2304890120

**Published:** 2023-05-08

**Authors:** Péter Németh, Laurence A. J. Garvie

**Affiliations:** ^a^Institute for Geological and Geochemical Research, Research Centre for Astronomy and Earth Sciences (MTA Centre of Excellence, Eötvös Loránd Research Network), Budapest H-1112, Hungary; ^b^Department of Earth and Environmental Sciences, University of Pannonia, Veszprém 8200, Hungary; ^c^Buseck Center for Meteorite Studies, Arizona State University, Tempe, AZ 85287-6004

Lonsdaleite is the mineral name given to the hexagonal diamond component in the hard carbon grains from the Canyon Diablo iron meteorite (1). Although natural and synthetic materials with diffraction data matching the Canyon Diablo lonsdaleite have been widely reported, recent studies showed that the structure of the hard carbon grains from the Canyon Diablo meteorite corresponds to a nanocomposite of c/h stacking disordered diamond and diaphite consisting of graphene layers coherently bonded to the cubic diamond matrix (2, 3). These studies demonstrated that nanostructured elements are intimately intergrown giving rise to structural features erroneously associated with hexagonal diamonds (2, 3).

It was recently reported that many ureilites, which are primitive achondrite meteorites, contain abundant micron-sized lonsdaleite grains (4). The identification was based on selected-area electron diffraction (SAED) data and cathode luminescence (CL) measurements, with supplementary data based on electron energy-loss spectroscopy (EELS) and X-ray diffraction (4). However, the lonsdaleite identification is not supported by the data. Most importantly, all of the SAED data can be interpreted as either graphite or cubic diamond, both of which were shown to exist in the sample (4), or diaphite, a material consisting of crystallographically associated diamond and graphene units (5). The fitting of the SAED data with a commercial software package is qualitative and does not provide proof of the presence of lonsdaleite.

Within the experimental measurement error (±1 to 2%), all the SAED data are consistent with graphite, cubic diamond, and diaphite. A major concern is that the sample was tilted, but not so as to show the diagnostic <010> projection and the discrete *h*0*l* reflections from which lonsdaleite identification would be unquestionable. The EELS data provide information on the bonding, but the data are inappropriate to distinguish between lonsdaleite and cubic diamond and do not exclude the occurrence of some graphitic material, both of which are identified. The X-ray diffraction pattern is also inconclusive as all the peaks attributed to lonsdaleite overlap with cubic diamond, goethite, and olivine.

A further issue relates to the interpretation of the CL data. The paper associates the 2.317 eV CL signal with lonsdaleite and based on this assignment maps the distribution of lonsdaleite. However, the paper does not cite references to support the 2.317 eV-lonsdaleite association. Previous work associates the 2.317 eV (535 nm) CL signal to clusters of N-doped diamonds (6).

It is concerning that paper (4) does not provide diffraction evidence for lonsdaleite in ureilite meteorites and fails to demonstrate why a CL signal reported for N-containing diamond (6) should be associated with lonsdaleite. The proposed formation of “lonsdaleite” and diamond in ureilite reported in ref. 4 is questioned.
